# Impact of screening between the ages of 60 and 64 on cumulative rates of cervical cancer to age 84y by screening history at ages 50 to 59: A population-based case-control study

**DOI:** 10.1016/j.ypmed.2021.106625

**Published:** 2021-08

**Authors:** Alejandra Castanon, Leonardo I. Green, Peter Sasieni

**Affiliations:** Faculty of Life Sciences & Medicine, School of Cancer & Pharmaceutical Sciences, Cancer Prevention Group, Innovation Hub, Guys Cancer Centre, Guys Hospital, Great Maze Pond, London SE1 9RT, UK

**Keywords:** Cervical cancer, Cervical screening, Older women, Screening over age 65, Cumulative risk, Population rates, Screening older women, HPV, Human papillomavirus, GP, General practitioner, RR, Relative risk, PY, Person years, NHS, National Health Service

## Abstract

There is little empirical data on the absolute benefit of cervical screening between ages 60-64y on subsequent cancer risk. We estimate the incidence of cervical cancer up to age 84y in women with and without a cervical cytology test at age 60-64y, by screening histories aged 50-59y. The current study is a population based case-control study of women born between 1928 and 1956 and aged 60-84y between 2007 and 2018. We included all such women diagnosed with cervical cancer in England and an aged-matched random sample without cancer. Women with a hysterectomy were excluded. Exposure was cervical cytology between ages 50–64y. The main outcome was 25y cumulative risk of cervical cancer between ages 60-84y. We found that eight in every 1000 (8.40, 95%CI: 7.78 to 9.07) women without a screening test between age 50‐64y develop cervical cancer between the ages of 60-84y. The risk is half: 3.46 per 1000 (95%CI: 2.75 to 4.36) among women with a test between age 60-64y but no cervical screening test at age 50-59y. The absolute difference in risk is equivalent to one fewer cancer for every 202 such women screened. The highest risk (10.01, 95%CI:6.70 to 14.95) was among women with abnormal screening at ages 50-59y and no tests 60-64y. 25y risk among women with a screening test every five years between age 50–64y was just under two in a 1000 (1.59, 95%CI:1.42 to 1.78). Results suggest the upper age of screening should be dependent on previous screening participation and results.

## Highlights

• Upper age of cervical screening is largely based on expert opinion and modelling.
• Women with a screening test every five-years between age 50–64y have a low subsequent risk of cervical cancer.
• Those with abnormal tests aged 50-59y and no test 60-64y have the highest risk (10 in 1000).
• For women with no test at age 50-59y, one test between age 60-64y halves the subsequent risk from 8.4 to 3.5 in 1000.
• The upper age of screening should be dependent on previous screening participation and results.

## 1 Introduction

In most high-income countries, adult women are encouraged to attend cervical screening regularly, but are discouraged from further screening once they reach their mid-60s. Although there is still some uncertainty regarding the optimal age to stop cervical screening, most studies suggest that for women who have tested negative when screened in their 50s or early 60s the risk of cervical cancer thereafter is low enough to question the need for screening over age 65y. Nevertheless, the upper age of cervical screening is largely based on expert opinion (Saslow et al., 2012; Gravitt et al., 2018) and modelling (Malagon et al., 2018) rather than empirical evidence. Further, studies of cervical screening with mortality as an endpoint support screening after age 65y to reduce deaths thereafter. (Lonnberg et al., 2013; Rustagi et al., 2014)

A study among women enrolled in Kaiser Permanente of Northern California (Landy et al., 2020) found that it is safe for women to exit screening after a negative co-test (i.e. both cytology and HPV test negative) between ages 55-64y, but the authors warn about generalising results to women whose only ever screen was a co-test at age 55y. A Swedish study (Wang et al., 2017) concluded that a cervical cytology at ages 61-65y reduced the subsequent risk of cervical cancer in women who had not been screened or who had abnormal tests in their 50s. However, among those who had negative screening results in their 50s, the extra benefit from another test between the ages of 61 and 65y was limited. A previous publication from England (Castanon et al., 2014) concluded that the risk of cervical cancer after age 65y remained very low for 5–10 years following a negative test; but by 15 years the risk had increased to half of that in never screened women.

Here the aim is to explore the impact of a cytology screening test between the ages of 60 and 64y (in women with different screening histories aged 50-59y) on cervical cancer incidence between ages 60 and 84y.

## 2 Methods

The NHS Cervical Screening Programme in England invites women aged 25-49y for screening by cytology every three years and women aged 50-64y every five years. Cervical cytology over age 65 is rare representing 0.8% of all tests taken in England. (Health and Social Care Information Centre, 2013) Cytology based organised cervical screening was introduced in England in 1988, HPV triage of low-grade cytological abnormalities was introduced in 2012. Between 2013 and 2019, there was a pilot of HPV primary testing in which about 5% of women nationally were screened by primary HPV testing with cytology triage or HPV genotyping. HPV primary testing was implemented nationally at the end of December 2019.

Since April 2007, there has been a national cervical screening audit in England. (NHS Cervical Screening Programme, 2006) Data on screening histories were abstracted from routinely recorded cervical cytology records held on the Cervical Screening Call/Recall System. This system includes all NHS (and many private provider) smears taken in England since 1988. The following information was available for each test: date, results and action taken in response to the test result (i.e. routine recall, early recall or referral). It does not include information on potential confounders such as smoking or ethnicity. The data used here were taken from the November 2019 anonymised dataset released for research purposes by Public Health England's Office for Data Release.

### 2.1 Study population

In this study, cases were women diagnosed with a primary invasive cervical cancer in England between April 2007 and March 2018 at ages 60-84y who were registered with an NHS general practitioner (GP). Eligible controls were all other women registered with an NHS GP at the time of a case's diagnosis and not known to have had a total hysterectomy. Two controls were individually matched on date of birth (within 2 years) and local geography to each case. Controls were randomly selected (using a computer program) from women who satisfied the matching criteria. Data were extracted on all selected controls whether or not they were screened. For analysis, controls were assigned the age of diagnosis of their matched case.

Prior to 1988, many women were screened opportunistically but those tests may not have been retrospectively logged in the Cervical Screening Call/Recall System and hence would not be recorded in the study dataset. To account for the possibility of misclassification when looking at recorded screening between the ages of 50-59y: women born prior to 1928 (i.e. age 61 or over in 1988) were excluded; and women born between 1928 and 1937 who had no recorded test were considered separately.

### 2.2 Classification of screening exposure

We define a negative test as a cytology test with a normal result (or a negative primary HPV test) after which a routine recall interval was recommended. Abnormal screening includes cytology results of borderline dyskaryosis or worse; inadequate with colposcopy recommended; and normal with a recommendation of a repeat test at a shorter interval than routine. Inadequate test results after which a repeat screening test was recommended were ignored when classifying women in screening exposure groups.

To examine the impact of screening at ages 60-64y, women born since 1938 were classified into four groups depending on screening history (including the test results) between the ages of 50-59y:

i) No recorded screens between ages 50-59y.
ii) Any abnormal result between 50-59y.
iii) Irregularly negatively screened: (no abnormal tests aged 50-59y and) a negative test between aged 50-54y but not 55-59y, or aged 55-59y but not 50-54y.
iv) Well screened with at least one negative result both at ages 50-54y and 55-59y (and no abnormal screen aged 50-59y).

Due to the lack of data on screening aged 50-54y in women born between 1928 and 1937, two additional groups were used (Supplementary Materials).

Screening exposure between the ages of 60-64y was defined as:

a) Screened – women with a screen which did not occur within six months of case-diagnosis (to exclude diagnostic tests),
b) Not screened – women with no test at ages 60-64y or if the only recorded test was within six months of diagnosis.

The combination of both categorisations resulted in eight screening history groups between the ages of 50–64y (plus four more for women born in 1928–1937).

To establish the effect of participating in screening between 50–64y inclusive and regardless of test results, women were classified as:

i) Never screened – defined as women with no test at ages 50–64y.
ii) Irregularly screened – defined as test(s) between the ages of 50–64y (but not in all three age-bands).
iii) Regularly screened – at least one test in each five-year age-group: 50-54y, 55-59y and 60-64y.

### 2.3 Data analysis

To estimate relative risks, controls were weighted to be representative of the population at risk. Cases were given a weight of one. Since women were not eligible as controls if they were known to have a hysterectomy, the number of women estimated to have had a hysterectomy was subtracted from mid-year population estimate to obtain the size of the population at risk. (Park, 2018) Hysterectomy prevalence percentages (projected for 2001–2010 and 2011–2012) in 5 year age bands were taken from published research. (Redburn and Murphy, 2001) Projections were based on hysterectomy rates in 1995. Since the number of hysterectomies performed in England fell between 1995 and the early 2000s, (Mukhopadhaya and Manyonda, 2013; Reid and Mukri, 2005) a sensitivity analysis was performed to assess the effect of a ± 10% change in hysterectomy prevalence on cervical cancer rates. A ± 10% relative change in the prevalence of hysterectomies had an average ± 3% (range 2.3% to 3.4%) relative impact on the absolute risks.

For each year of diagnosis and age group, the control weights were calculated using the following formula:

$$\text{Control weight}=\frac{\left(\;\text{ratio of cases to controls}\right)}{\left(\text{incidence rate in women with}\;a\;\text{uterus}\right)}-\left(\text{ratio of cases to controls}\right)$$

Cervical cancer incidence rates in women with a uterus were calculated as the number of cases diagnosed nationally (Office for National Statistics, 2021) divided by the hysterectomy-adjusted mid-year population estimate.

When considering screening histories (12 categories) separately in each 5-year age-group we were concerned that the small number might lead to logically inconsistent estimates of risk. Constraints were imposed to ensure that more intensive screening aged 50-59y was not associated with higher absolute risk of cervical cancer (in each age band separately for screened and unscreened at age 60-64y). Partial orderings were defined so that the estimated absolute risk in women with less intensive screening was equal or greater than that in women with more intensive screening; and abnormal screening had greater risk than irregular screening (supplemental material and Table S1). Comparison of models can be found in Table S2. We used a weighted relative risk (RR) regression model including interactions between screening aged 50–64y (12 levels for screening history) and age at diagnosis (60-64y, 65-69y, 70-74y, 75-79y and 80-84y). The inverse probability of sampling weights allowed estimation of women-years at risk in the population, annual rates (absolute risk), and relative risks. Cumulative risks at ages 60-84y are the sum of the age specific risks, 95% CI were obtained in STATA and are presented per 1000 women.

When exploring risk by screening participation no adjustments were necessary (i.e., the constrained and unconstrained model fits were identical). Cumulative risks at ages 65-84y and their 95% confidence intervals are presented per 1000 women.

To explore differential opportunities for screening (opportunity bias), we also present these results restricted to the control matched on GP practice. We estimate risks to age 84y because women aged 65y in England (in 2015–2017) are expected to live a further 20.9 years. (Office for National Statistics, 2018)

We use population attributable factions to estimate the impact on population cervical cancer rates over the next 25-years of screening women once (more) between the ages of 60-64y.

• Since the data in this study are from a matched case-control design, the distribution of screening histories in cases were used to represent the distribution in all women with cervical cancer. To estimate the proportion of the population in each screening history category the proportion of cases diagnosed age 65-69y in that category were divided by the relative risks for that screening history category.
• The difference in 25y absolute risks between those screened and unscreened at ages 60-64y by screening history age 50-59y was multiplied by the estimated proportion of women in the population to obtain the impact of screening women at ages 60-64y on rates of cervical cancer thereafter.
• Cumulative rates are presented per 1000 women in the English population.

## 3 Results

**Fig. 1:**
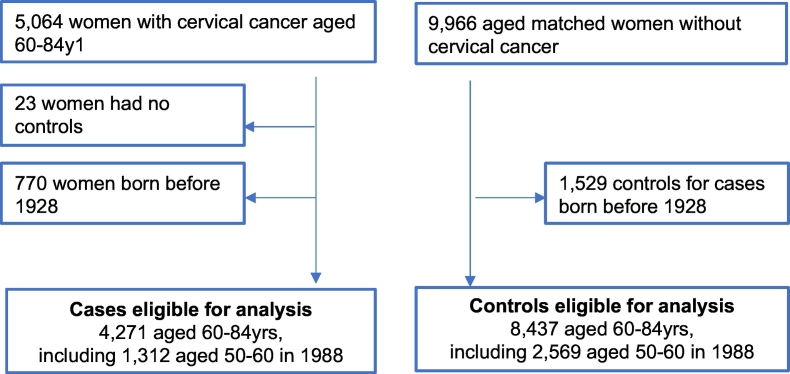
Flowchart detailing eligibility for analysis.

A total of 4271 women with cervical cancer diagnosed between the ages of 60-84y and 8437 age-matched women without cervical cancer were eligible for analysis corresponding to 34.9 million women-years of follow-up for cancer (in 2007–2018) (Table 1). The women in this study comprise 85% of cervical cancers diagnosed in England among women aged 60 to 69y, 74% in women aged 70-79y and 70% in women aged 80-84y. In the study, 31% of women with cervical cancer were aged 50-60y in 1988 when the organised screening programme was first introduced and would not have been invited within the programme in their 30s nor 40s. A further 44% were eligible for screening since their 40s (i.e., aged 40–50 in 1988). Fig. 1 details the study inclusion criteria.

**Table 1:** Characteristics of the study population.

	Cases		Controls			Cervical cancer rate per 100,000
	N	%	N	%	PY^a^ 1000's	
Total	4271	100%	8437	100%	34,756	12.3
Birth cohort						
1928 to 1937	1312	31%	2596	31%	8906	14.7
1938 to 1947	1886	44%	3733	44%	16,446	11.5
1948 to 1956	1073	25%	2108	25%	9404	11.4
Age at diagnosis						
60-64y	1254	29%	2477	29%	10,791	11.6
65-69y	1000	23%	1973	23%	9049	11.1
70-74y	804	19%	1583	19%	6672	12.1
75-79y	817	19%	1623	19%	5574	14.7
80-84y	396	9%	781	9%	2671	14.8
Screening status between age 60-64y						
Screened	1528	36%	4885	58%	19,939	7.7
Not screened	2743	64%	3552	42%	14,817	18.5
Screening history between age 50-59y						
None (born from 1938)	1424	33%	880	10%	3880	36.7
None (born 1928 to 1937)	573	13%	720	9%	2372	24.2
Abnormal screening	437	10%	450	5%	1898	23.0
Irregular screening	409	10%	912	11%	3921	10.4
One screen age 55-59y (born 1928 to 1937)	305	7%	733	9%	2483	12.3
Well screened	1123	26%	4742	56%	20,202	5.6
Screening participation						
No screening	1856	43%	1309	16%	5267	35.2
Screened irregularly	1445	34%	3538	42%	14,472	10.0
Screened regularly	970	23%	3590	43%	15,169	6.4

a Person years.

The age distribution was skewed relative to the age distribution of cervical cancer each year due to eligibility criteria, Table 1. There were just 18 HPV primary screening tests, all of which were negative for HPV. Crude rates of cervical cancer decreased with increasing screening intensity regardless of the definition of screening exposure, Table 1.

### 3.1 Impact of screening aged 60-64y on 25 year-risk of cervical cancer by screening history aged 50-59y

The risk of cervical cancer was greatest in those with a previous abnormal screen age 50-59y and no tests between ages 60-64y; 10.01 in every 1000 developed cervical cancer over 25-years. Among those with an abnormal screen aged 50-59y, the difference in risk over 25-years between women screened aged 60-64y and women not screened aged 60-64y was 4.34 per 1000 women, equivalent to one fewer cancer for every 230 women screened (Table 2).

**Table 2:** 25-year absolute risk and difference in risk per 1000 women.

Screening history age 50-59y	Screening status age 60-64y	Risks are per 1000 women with a given screening history					Reduction in the 25-year rate of cervical cancer per 1000 population aged 60-84y associated with screening at ages 60-64y			
		Absolute 25-year risk per 1000 women with a cervix	Difference in 25-y risk per 1000 for screening 60-64y	NNS^a^ to prevent one cervical cancer	% of women in each screening category	25-year risk per 1000 in England	Achieved in women with a cervix	Achieved at population level	Achievable in women with a cervix, if all screened	Achievable at population level if all screened
No Screening	None	8.40 (7.78, 9.07)	4.94 (3.92, 5.96)	202 (168, 255)	20.7%	1.74			1.02 (0.81, 1.23)	0.80 (0.81, 0.96)
	Screened	3.46 (2.75, 4.36)			2.0%	0.07	0.10 (0.08, 0.12)	0.08 (0.06, 0.09)		
Abnormal screening	None	10.01 (6.70, 14.95)	4.34 (0.11, 8.57)	230 (117, 9091)	1.2%	0.12			0.05 (0.00, 0.10)	0.04 (0.00, 0.08)
	Screened	5.67 (4.48, 7.18)			4.1%	0.23	0.18 (0.01, 0.35)	0.14 (0.00, 0.28)		
Irregular screening	None	3.62 (3.10, 4.21)	1.29 (0.65, 1.93)	775 (518, 1538)	7.9%	1.14			0.10 (0.05, 0.15)	0.08 (0.04, 0.12)
	Screened	2.33 (2.02, 2.68)			5.3%	0.12	0.07 (0.04, 0.10)	0.05 (0.03, 0.08)		
Well screened	None	2.22 (1.71, 2.87)	0.63 (0.03, 1.23)	1587 (813, 37,037)	9.8%	0.22			0.09 (0.03, 0.14)	0.07 (0.02, 0.11)
	Screened	1.59 (1.42, 1.78)			49.0%	0.78	0.43 (0.14, 0.72)	0.33 (0.11, 0.56)		
Population average					100%	3.58^b^				
Total benefit							0.77 (0.26, 1.29)	0.60 (0.20, 1.01)	1.26 (0.89, 1.63)	0.99 (0.70, 1.27)

Impact of one screen between ages 60-64y by screening history aged 50–64y on rates of cervical cancer over 25-years at a population level and among women with a cervix.

a Number needed to screen (NNS).

b We found the annual risk per 100,000 women to be 14.3 ((3.58/25)*100). Note that the proportion of women who are unscreened between ages 50 and 64 has increased in recent years. If we had used the screening history distribution of cases diagnosed at age 75–79 (instead of 65-69y), the 25-year risk per 100,000 women with a cervix would be 12.4 which is very similar to 12.1 the average estimated from the national cancer statistics.

8.40 in a 1000 (95%CI: 7.78–9.07) women without a screening test between age 50–64y developed cervical cancer over a 25-year period (Table 2). Women with no tests age 50-59y but with a single test between age 60 and 64y had a risk of 3.46 per 1000 (95%CI: 2.7–4.36). The absolute difference in risk is equivalent to one fewer cancers for every 202 such women screened.

Among well-screened women with a test at ages 60-64y (i.e., those with a negative screening test both age 50-54y and 55-59y and no abnormal screen aged 50-59y), 1.59 per 1000 (95%CI: 1.42–1.78) developed cervical cancer over the next 25-years (Table 2). Having an extra test aged 60-64y in women well-screened (negatively) aged 50-59y was associated with one fewer cancer over the next 25y per 1587 women.

**Fig. 2:**
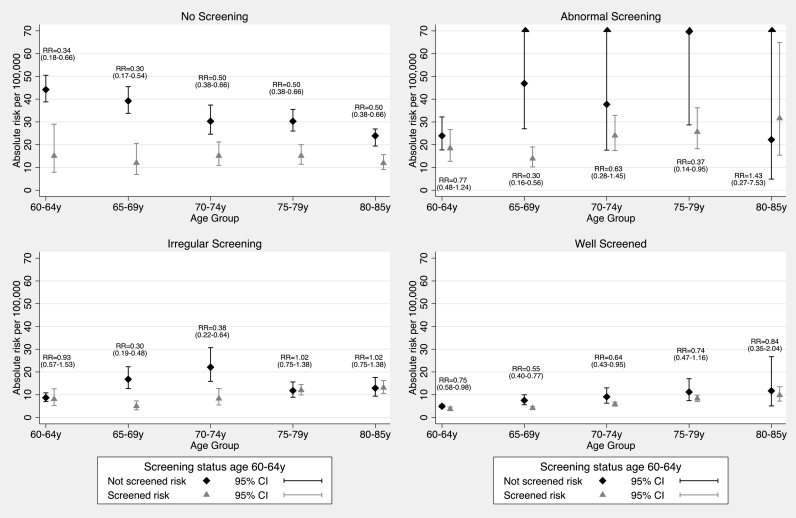
Annual risk (constrained model) per 100,000 women (with a cervix) by age, screening history age 50-59y and screening age 60-64y.Text within each graph reports relative risks (RR) and 95% confidence intervals in parenthesis.

Analysis restricted to case-controls pairs matched on GP practice as well as age yielded similar results (Table S3). Results by age-group (Fig. 2) at diagnosis show the convergence of absolute risks with time since exiting screening at age 65y regardless of screening history age 50-59y.

### 3.2 Population impact of one screen between ages 60-64y

Under the assumption that the observed relative risks associated with screening are causal, the population impact of screening different groups of women currently aged 60-64y on cervical cancer rates (over 25-years) is presented in Table 2.

Current annual rates of cervical cancer in England at ages 60-84y are 9.3 per 100,000 women (Office for National Statistics, 2021) (equivalent to 12.1 per 100,000 women with a cervix). Projection of rates to the cohort of women with a cervix aged 65-69y in this study, suggest that their 25-year risk (from their 60th birthday) will be 3.58 per 1000. Screening at ages 60-64y since the year 2000 is projected to have reduced rates of cervical cancer in women aged 60-84y in England by an average of 0.60 per 1000 women (i.e., from 4.18 to 3.58 per 1000).

Cervical cancer rates in women with a cervix in England over the age of 60y could be further reduced by 1.26 per 1000 (from 3.58 to 2.32 or by 35%) if all those not currently screened aged 60-64y were screened once between the ages of 60-64y. More than three quarters (1.02 per 1000 or 28%) of this added benefit could be achieved by screening the 20.7% who have not attended screening since age 50y.

### 3.3 Impact of participation in screening

The 20-year risk of cervical cancer following five-yearly screening between ages 50–64y was 1.60 in 1000 women (95%CI: 1.43–1.79); this risk increased to 2.55 in 1000 (95%CI: 2.35–2.76) for women who were screened irregularly between ages 50–64y. (Table 3). The risk in women not screened between ages 50–64y was 6.16 in 1000 (95%CI: 5.64–6.72). These results support a dose-response (with screening intensity), i.e. a lower risk of cervical cancer with increased intensity of screening. Further, both the relative and absolute differences in risk decrease with increasing age (and increasing time from last screen).

**Table 3:** Participation in screening.

Screening participation age 50–64y	Aged 65-69y			Aged 70-74y			Aged 75-79y			Aged 80-84y			Aged 65-84y	
	Cases (N)	PY^a^ 1000's	Risk per 1000 PY^a^ (95% CI)	Cases (N)	PY^a^ 1000's	Risk per 1000 PY^a^ (95% CI)	Cases (N)	PY^a^ 1000's	Risk per 1000 PY^a^ (95% CI)	Cases (N)	PY^a^ 1000's	Risk per 1000 PY^a^ (95% CI)	20-year cumulative absolute risk per 1000 PY^a^	20-year difference^b^ in absolute risk per 1000 PY^a^ (95% CI)
No screening	485	1237	0.39 (0.34, 0.46)	295	1007	0.29 (0.25, 0.35)	295	975	0.30 (0.26, 0.36)	134	548	0.24 (0.19–0.31)	6.16 (5.64, 6.72)	–
Screened irregularly	249	2255	0.11 (0.10, 0.13)	223	1632	0.14 (0.12, 0.16)	294	2195	0.13 (0.12, 0.15)	202	1559	0.13 (0.11–0.15)	2.55 (2.35, 2.76)	3.61 (3.08, 4.23)
Screened regularly	266	5557	0.05 (0.04, 0.06)	286	4032	0.07 (0.06, 0.08)	228	2403	0.10 (0.08, 0.11)	60	564	0.11 (0.08–0.14)	1.60 (1.43, 1.79)	4.56 (4.02, 5.16)

Annual and 20-year cumulative risk of cervical cancer per 1000 women (with a cervix) by age at diagnosis and screening participation age 50–64y. Constrains were not necessary.

a Person years.

b Relative to “no screening”: Amount by which risk is lower.

## 4 Discussion

Screening intensity prior to age 65y is prognostic of risk thereafter. Women with regular screening between the ages of 50–64y are at low risk of cervical cancer up to age 84y. However, those who have not been screened at ages 50–64y (or those with positive screening tests) are at high risk of developing cervical cancer.

Regardless of screening history at ages 50-59y, women who attend aged 60-64y are at lower risk thereafter. Whilst acknowledging that the magnitude of the impact of screening estimated here could be affected by confounding, we interpret this association as causal partly because there is a substantial body of evidence suggesting that cervical screening can greatly reduce the risk of cervical cancer (IARC, 2005).

Since the underlying risk is so much greater in those not screened aged 50–59, the absolute benefit of a screen at ages 60-64y is eight times greater among women who were unscreened age 50-59y than among those who were well screened and had a test age 60-64y. Without screening about 8.4 per 1000 women (with a cervix) in England currently aged 60-64y would get cancer by age 84y, but a single test between aged 60-64y would prevent 4.9 of those 8.4 cancers. Five-yearly screening from age 50–64y (i.e., three screens) would prevent about 6.8 (i.e., 8.40 minus 1.59) of those cancers.

A reduction in cervical cancer rates of 1 per 1000 women years could be achieved just by screening the 20.7% of women (with a cervix) who have not attended screening since age 50y once between ages 60-64y. If efforts were made to screen previous non-attenders once in their early 60s, the cumulative effect over the next 40 years would be substantial.

A similar study from Sweden (Wang et al., 2017) estimated 20y cumulative incidence to age 80y using a nationwide cohort. In well-screened women (aged 50–64y) incidence was 0.13%, similar to that observed in this study (0.16%) to age 84y. The cumulative incidence in unscreened women (no test age 51-65y) was lower in the Swedish study (0.50%) than observed here (0.84%). There are several plausible explanations for the differing result. The unadjusted 20y risk in unscreened women was 0.62% in this study suggesting results may not be substantially different. Alternatively, the underlying incidence of cervical cancer could have been lower in Sweden than in England.

Prior research looking at the impact of screening on mortality in older women has found lower risk of death among those screened prior to diagnosis. (Lonnberg et al., 2013; Rustagi et al., 2014; Pankakoski et al., 2019) In the UK only 18% of cervical cancers occur age 65 and over, whereas 47% of deaths occur in this age group. (Cancer Reaserch UK, 2020) Hence there is a clear benefit in screening older women to either prevent or diagnose cervical cancer earlier (Landy et al., 2015) ensuring better survival.

The highest risk of cervical cancer after age 60y was among those who have abnormal test results (in their 50s) for whom the risk remained high for 25-years. A single additional screen aged 60-64y substantially reduces this risk, but it remains high and continued surveillance maybe warranted for this population.

Results presented here reflect cytology-based screening that happened between 4 and 32 years ago. Some have cautioned against extrapolating past risk experience (especially in post-menopausal women) to today's population. (Gravitt et al., 2018) Extrapolating the risk of cervical cancer in unscreened women in this study (0.84% from age 60-84y) backwards, we estimate the lifetime risk in unscreened women (from age 20-84y) to be 2.2% which reinforces the value of screening women aged 20 to 50y. This lifetime risks in unscreened women is similar to that reported in the literature for England. (Landy et al., 2018; Cancer Research UK, 2016)

Absolute risk following one or more negative HPV tests may be much lower than those reported here. According to a recent Canadian study (Malagon et al., 2018) a woman who has participated in screening until age 55y and has a negative HPV test at age 55y, has about a 0.05% chance of getting cervical cancer after the age of 55y. This risk is sufficiently small as to make it difficult to justify further screening. What it does suggest is that risks reported here in screened women could potentially be halved following the introduction of HPV primary testing; and the benefits of screening previously unscreened women would be even greater.

We have estimated absolute risks among women screened with cytology using a case-control design. The population-based study design means that we have information on over three-quarters of cancers diagnosed in England in women aged 60-84y. Obtaining screening data from the call/recall database eliminated recall bias. Hysterectomy is one of the most common surgical procedures with about 60,000 performed per year in England, (Redburn and Murphy, 2001) so we have adjusted absolute risks for hysterectomy rates. The risk of cervical cancer varied by birth cohort. Matching closely on birth date will have mitigated against this bias. By matching cases and controls on small-area geography and date of birth we have minimised the bias arising from differential opportunities for screening.

Confounding due to social and behavioural factors could remain, in particular the fact that those who are at lower underlying risk of cervical cancer are also most likely to attend screening (self-selection bias). We have no data with which to make adjustments for such a bias. However the magnitude of the observed effects, the monotone dose response (with intensity of screening), the converging absolute risk with time since exiting screening at age 65y and the similarity between the estimated effect of screening using individual level exposure here (66% lower risk in screened vs unscreened at age 60–64, Fig. 2) and those estimated from trends at a population level (57% reduction in incidence rates) (Cancer Reaserch UK, 2018) following the introduction of screening, support the notion that risks observed over age 65 are largely due to screening.

There is a posibiliy of misclasification of screening histories, particulary among those who were aged 50 or over in 1988 since any screening they received prior to this may not have been recorded in the call/recall system. Imposing contraints will have limited the impact of this bias on the results. The constraints greatly reduced the variance in the absolute risks at older ages where data was sparce without significantly changing the overall goodness of fit.

We have assumed that the inclusion of women into the dataset was independent of their screening history. However, if their inclusion were related to screening the bias would most likely be against screening. For example, we conjecture that cancers missed from the audit are more likely to be those diagnosed too late for treatment and that women who present very late are less likely to have participated in screening in their 50s and 60s.

Relative reductions in risk of cervical cancer reported in this study are likely to be generalisable to other setting with good quality screening, whereas absolute rates are particular to England.

Our study adds to the growing literature informing the debate on approriate upper ages to discontinue screening and in particular to the suggestion that the upper age of screening should be dependent on previous screening participation and results. It benchmarks impact of cytology based cervical screening allowing for comparisons to HPV testing in the future and provides data to underpin modelling of the cost- effectiveness of screening women beyond age 65y.

## 5 Conclusion

Results suggest the upper age of screening should be dependent on previous screening participation and results. Ultimately decisions on when to cease screening will depend on societies attitude to risk and available resources. However, given the low lifetime risk, it is safe for women with three negative tests after age 50y to exit cervical screening at age 65y.

## Ethics approval

Ethical approval was obtained from the Liverpool Central Research Ethics Committee (ref 17/NW/0655). Data for this study are routinely collected as part of the audit of invasive cervical cancer, anonymised and quality assured by the PHE Population Screening Programmes. Access was facilitated by the PHE Office for Data Release (ODR1718_424).

## Role of the funding source

This work was supported by a 10.13039/501100000289Cancer Research UK programme grant [grant number C8162/A27047]. The funder had no role in the preparation of the manuscript or the decision to submit for publication. All authors had full access to the data in the study and accept responsibility to submit for publication.

## Data sharing statement

Data is available upon request to the Public Health England Office for Data Release.

## Declaration of Competing Interest

The authors declare that they have no known competing financial interests or personal relationships that could have appeared to influence the work reported in this paper.

## Appendix A Supplementary data

Supplementary material Image 1
