# Supplementary material for: Impact of screening between the ages of 60 and 64 on cumulative rates of cervical cancer to age 84y by screening history at ages 50 to 59: A population-based case-control study
Source: Prev Med. 2021 Aug;149:106625. doi: 10.1016/j.ypmed.2021.106625 (PMC8223500; doi:10.1016/j.ypmed.2021.106625)
Supplement: Supplementary file 1 — Supplementary material [file mmc1.docx]

**Supplementary materials**

**Model constrains and partial ordering**

Whereas the unconstrained models (with either 15 or 60 parameters) provide unbiased estimates of the rates in each subgroup, some of the estimates will have large variances and are uninformative. For this reason we imposed constraints to ensure that more intensive screening was not associated with higher absolute risk of cervical cancer (in each age band separately for screened and unscreened).

Partial orderings were defined so that the absolute risk in the categories of screening with less intensive screening had equal or greater risk to more intensive screening categories; and abnormal screening had greater risk than irregular screening (supplementary table 1).

- None ≥ Irregular screening ≥ Regular screening
- None (born after 1938) ≥ None (born 1928 to 1938) ≥ Irregular screening ≥ Well screened negative
- None (born 1928 to 1938) ≥ One screen age 55-59y (born 1928 to 1938) ≥ Well screened negative
- Abnormal screening ≥ Irregular screening ≥ Well screened negative.
- Abnormal screening ≥ One negative screen age 55-59y (born 1928 to 1938) ≥ Well screened negative.

Additionally, for each screening history age 50-59y, the relative risks associated with screening age 60-64y (yes/no) were constrained to prevent the relative risks after age 65y getting smaller with increasing age. That is, we assured that any protective effect was attenuated (if it changed at all) with increasing time since the screen (i.e. from age 65 onwards). Such as decision was taken following evidence that risk of cervical cancer attenuates with time since last test in women over age 64y.(1)

When any of these order constraints were violated, the model was refit forcing parameters to be equal in violating categories (detailed in supplementary table 2). To satisfy the a priori ordering, we imposed nine equality constraints (reducing the number of parameters from 60 to 51), supplementary table 2. Nested models were compared using Bayesian information criteria (BIC), Akaike Information Criterion (AIC). Both the BIC and the AIC led to the selection of the constrained model; the reduction in goodness of fit associated with imposing constraints was not significant, chi2(9)= 6·89, P=0·65.

Although potentially controversial, we feel that imposing these natural ordering constraints has resulted in a flexible yet realistic model. We also note that the constraints make little difference to the point estimates of the cumulative risk of cervical cancer beyond aged 65y

**Reference**

1. Castanon A, Landy R, Cuzick J, Sasieni P. Cervical screening at age 50-64 years and the risk of cervical cancer at age 65 years and older: population-based case control study. PLoS medicine. 2014;11(1):e1001585.

Table S1. Summary of equity constraints imposed on the model

| **Constraints for the absolute risks** | | | | | | |
| --- | --- | --- | --- | --- | --- | --- |
| None -born after 1938 (age 75-79y) | = | None - born 1928 to 1938  (age 75-79y) | | | | |
| Irregular  (age 75-79y) | = | One screen 55-59y - born 1928-1938  (age 75-79y) | | | | |
| Irregular  (age 80+y) | = | One screen 55-59y - born 1928-1938  (age 80+y) | | | | |
| **Constraints for the relative risks (unscreened aged 60-64 as baseline)** | | | | | | |
| None -born after 1938 (age 70-74y) * | = | None -born after 1938  (age 75-79y) | = | None - born 1928 to 1938  (age 75-79y) | = | None - born 1928 to 1938  (age 80+y) |
| Irregular  (age 75-79y) ^£^ | = | Irregular  (age 80+y) | = | One screen 55-59y - born 1928-1938  (age 75-79y) | = | One screen 55-59y - born 1928-1938 (age 80+y) |

* ^£^  Indicate equity constraints on the relative risks. The constraints for the absolute risks are shaded in supplementary table 2.

Table S2. Absolute and relative risk of cervical cancer from the unconstrained and constrained models by age at diagnosis, screening history age 50-59y and screening status age 60-64y.

|  |  | **Unconstrained** | **Constrained** |
| --- | --- | --- | --- |
| **Screening history aged 50-59y** | **Age-group** | **Absolute risk (per 100,000 PY) if not screened age 60-64** | |
| None (born from 1938) | 60-64 | 44·2 | 44·2 |
| None (born 1928 to 1937) | 60-64 | - | - |
| Abnormal screening | 60-64 | 23·9 | 23·9 |
| Irregular screening | 60-64 | 8·7 | 8·7 |
| One screen age 55-59y (born 1928 to 1937) | 60-64 | - | - |
| Well screened negative | 60-64 | 4·9 | 4·9 |
| None (born from 1938) | 65-69 | 39·2 | 39·2 |
| None (born 1928 to 1937) | 65-69 | 28·0 | 28·0 |
| Abnormal screening | 65-69 | 46·9 | 46·9 |
| Irregular screening | 65-69 | 16·8 | 16·8 |
| One screen age 55-59y (born 1928 to 1937) | 65-69 | - | - |
| Well screened negative | 65-69 | 7·5 | 7·5 |
| None (born from 1938) | 70-74 | 30·1 | 30·3 |
| None (born 1928 to 1937) | 70-74 | 27·8 | 27·8 |
| Abnormal screening | 70-74 | 37·7 | 37·7 |
| Irregular screening | 70-74 | 22·1 | 22·1 |
| One screen age 55-59y (born 1928 to 1937) | 70-74 | 13·5 | 13·5 |
| Well screened negative | 70-74 | 9·1 | 9·1 |
| None (born from 1938) | 75-79 | 28·5 | 30·3 |
| None (born 1928 to 1937) | 75-79 | 30·6 | 30·3 |
| Abnormal screening | 75-79 | 69·6 | 69·6 |
| Irregular screening | 75-79 | 11·8 | 11·8 |
| One screen age 55-59y (born 1928 to 1937) | 75-79 | 12·0 | 11·8 |
| Well screened negative | 75-79 | 11·2 | 11·2 |
| None (born from 1938) | 80-84 | - | - |
| None (born 1928 to 1937) | 80-84 | 24·4 | 23·9 |
| Abnormal screening | 80-84 | 22·2 | 22·2 |
| Irregular screening | 80-84 | 4·9 | 12·9 |
| One screen age 55-59y (born 1928 to 1937) | 80-84 | 15·7 | 12·9 |
| Well screened negative | 80-84 | 11·7 | 11·7 |
| **Screening history aged 50-59y** | **Age-group** | **Relative risk for screened aged 60-64y (relative to not screened aged 60-64y)** | |
| None (born from 1938) | 60-64 | 0·34 | 0·34 |
| None (born 1928 to 1937) | 60-64 | - | - |
| Abnormal screening | 60-64 | 0·77 | 0·77 |
| Irregular screening | 60-64 | 0·93 | 0·93 |
| One screen age 55-59y (born 1928 to 1937) | 60-64 | - | - |
| Well screened negative | 60-64 | 0·75 | 0·75 |
| None (born from 1938) | 65-69 | 0·30 | 0·30 |
| None (born from 1938) | 70-74 | 0·60 | 0·50 |
| None (born from 1938) | 75-79 | 0·22 | 0·50 |
| None (born from 1938) | 80-84 | - | - |
| None (born 1928 to 1937) | 65-69 | - | - |
| None (born 1928 to 1937) | 70-74 | 0·91 | 0·91 |
| None (born 1928 to 1937) | 75-79 | 0·55 | 0·50 |
| None (born 1928 to 1937) | 80-84 | 0·46 | 0·50 |
| Abnormal screening | 65-69 | 0·30 | 0·30 |
| Abnormal screening | 70-74 | 0·63 | 0·63 |
| Abnormal screening | 75-79 | 0·37 | 0·37 |
| Abnormal screening | 80-84 | 1·43 | 1·43 |
| Irregular screening | 65-69 | 0·30 | 0·30 |
| Irregular screening | 70-74 | 0·38 | 0·38 |
| Irregular screening | 75-79 | 0·85 | 1·02 |
| Irregular screening | 80-84 | 4·51 | 1·02 |
| One screen age 55-59y (born 1928 to 1937) | 65-69 | - | - |
| One screen age 55-59y (born 1928 to 1937) | 70-74 | 0·63 | 0·63 |
| One screen age 55-59y (born 1928 to 1937) | 75-79 | 1·02 | 1·02 |
| One screen age 55-59y (born 1928 to 1937) | 80-84 | 0·81 | 1·02 |
| Well screened negative | 65-69 | 0·55 | 0·55 |
| Well screened negative | 70-74 | 0·64 | 0·64 |
| Well screened negative | 75-79 | 0·74 | 0·74 |
| Well screened negative | 80-84 | 0·84 | 0·84 |
| *Blue and orange shading indicates where constrains have been imposed | | | |

Table S3. GP controls only. Absolute 25-year cumulative risk and risk difference per 1000 women with a given screening history (from the constrained model).

| **Screening history age 50-59y** | **Screening status age 60-64y** | **Cases N (%)^1^** | | **Controls**  **PY^2^ 1000’s (%)** | | **Absolute 25-year risk per 1,000 women with a cervix** | **Difference in absolute 25-year risk per 1,000** |
| --- | --- | --- | --- | --- | --- | --- | --- |
| No Screening | None | 1177 | 40% | 3861 | 16% | 8.10 (7.35, 8.92) | 5.09 |
|  | Screened | 124 | 4% | 1002 | 4% | 3.01 (2.32, 3.90) | (3.98, 6.20) |
| Abnormal screening | None | 74 | 2% | 117 | 0·5% | 12.78 (7.28, 22.42) | 6.98 |
|  | Screened | 221 | 7% | 1074 | 4% | 5.80 (4.48, 7.51) | (-0.36, 143.2) |
| Irregular screening | None | 242 | 8% | 1595 | 7% | 3.64 (2.99, 4.43) | 1.26 |
|  | Screened | 332 | 11% | 3206 | 13% | 2.38 (2.02, 2.81) | (0.45, 2.07) |
| Well Screened | None | 141 | 5% | 1656 | 7% | 2.10 (1.63, 2.70) | 0.50 |
|  | Screened | 664 | 22% | 11565 | 48% | 1.60 (1.41, 1.82) | (-0.07, 1.06) |
| ^1^ Note the smaller sample size. Not all cases had a GP control  ^2^ Person Years | | | | | | |  |
